# Genomic evidence for intraspecific hybridization in a clonal and extremely halotolerant yeast

**DOI:** 10.1186/s12864-018-4751-5

**Published:** 2018-05-15

**Authors:** Cene Gostinčar, Jason E. Stajich, Jerneja Zupančič, Polona Zalar, Nina Gunde-Cimerman

**Affiliations:** 1 Department of Biology, Biotechnical Faculty, University of Ljubljana, Ljubljana, Slovenia; 2 Department of Molecular and Biomedical Sciences, Jožef Stefan Institute, Ljubljana, Slovenia; 3 Department of Microbiology and Plant Pathology, Institute for Integrative Genome Biology, University of California-Riverside, Riverside, CA USA

**Keywords:** Whole genome duplication, Hybridization, Clonal reproduction, Restricted recombination, Halotolerance, Polyextremotolerance, Salt stress, Black yeast, Ploidy

## Abstract

**Background:**

The black yeast *Hortaea werneckii* (Dothideomycetes, Ascomycota) is one of the most extremely halotolerant fungi, capable of growth at NaCl concentrations close to saturation. Although dothideomycetous fungi are typically haploid, the reference *H. werneckii* strain has a diploid genome consisting of two subgenomes with a high level of heterozygosity.

**Results:**

In order to explain the origin of the *H. werneckii* diploid genome we here report the genome sequencing of eleven strains isolated from different habitats and geographic locations. Comparison of nine diploid and two haploid strains showed that the reference genome was likely formed by hybridization between two haploids and not by endoreduplication as suggested previously. Results also support additional hybridization events in the evolutionary history of investigated strains, however exchange of genetic material in the species otherwise appears to be rare. Possible links between such unusual reproduction and the extremotolerance of *H. werneckii* remain to be investigated.

**Conclusions:**

*H. werneckii* appears to be able to form persistent haploid as well as diploid strains, is capable of occasional hybridization between relatively heterozygous haploids, but is otherwise limited to clonal reproduction. The reported data and the first identification of haploid *H. werneckii* strains establish this species as a good model for studying the effects of ploidy and hybridization in an extremotolerant system unperturbed by frequent genetic recombination.

**Electronic supplementary material:**

The online version of this article (10.1186/s12864-018-4751-5) contains supplementary material, which is available to authorized users.

## Background

The black yeast *Hortaea werneckii* has been studied for more than two decades for its extreme halotolerance, which exceeds most other fungi. After its genome was sequenced it revealed another intriguing trait of the species: a diploid genome consisting of two (nearly) complete, but highly heterozygous genomes [1, 2]. Ploidy changes in fungi are often associated with environmental perturbations [3]. Considering that genomes of species related to *H. werneckii* are typically haploid, a possible link between the unusual diploid genome of *H. werneckii* and its extremotolerant biology was proposed, but not investigated further [2]. The divergence between the two *H. werneckii* subgenomes is relatively large, with only 89% of nucleotides conserved in regions that could be uniquely aligned between the two subgenomes. However, the expression of the paralogues was conserved to a high degree [2].

Reports of whole genome duplication in fungi remain scarce. They include examples from early diverging fungi [4, 5] and also from asco- and basidiomycetes [6]. The best known and also the most studied example is the ancient duplication in *Saccharomyces cerevisiae*, which was recently reported to have occurred through an ancient hybridization [7]. Interspecies hybrids are often asexual, but in the example of *Zygosaccharomyces parabailii* it was shown that hybrids can regain fertility by inactivating one mating-type locus [8].

Although a mating locus was identified in the reference *H. werneckii* genome, sexual reproduction has not yet been observed in this species [1]. However, the once widespread belief that up to a fifth of fungal species are strictly clonal was challenged by the increasingly powerful genomic and population genetic/genomic analyses [9].

In the absence of a large population dataset and with only one available *H. werneckii* genome sequence, the origin of the reference genome duplication could not be investigated. It was also unknown whether the diploid genome is a general trait of the species or just a peculiarity of the reference strain. When diploidy was first confirmed by sequencing the reference genome [1, 2], two explanations of its formation were discussed: an endoreduplication, and an intraspecific hybridization. Both MAT alleles of the reference strain identified at the time were MAT1–1 and the nucleotide sequence of the MAT genes was only 88.7% identical. Therefore endoreduplication was proposed as a preliminary hypothesis until further data became available [2]. Here we tested this hypothesis by sequencing eleven *H. werneckii* genomes and using comparative genomics. However, rather than providing evidence for endoreduplication, our results indicate the existence of not only one, but several hybridization events in the evolution of the twelve compared genomes.

## Results

In order to investigate the origin of the diploid genome of the reference strain of *H. werneckii*, we sequenced the genomes of additional eleven strains of the species (Table 1) selected to represent different intraspecific phylogenetic lineages based on a larger phylogenetic analysis of the species (data not shown) with no prior knowledge about their ploidy. After sequencing the genomes were compared to the reference genome, assembled and annotated.

**Table 1:** Strains of *Hortaea werneckii* compared in this study

Ex culture collection strain number	Name in this study	Isolation habitat and location of sampling site
EXF-2000	A	Hypersaline water; Sečovlje saltpans, Slovenia; reference genome [2]
EXF-120	B	Hypersaline water; Santa Pola saltpans, Spain
EXF-562	C	Soil on the sea coast; Namibia
EXF-2788	D	Hypersaline water; Sečovlje saltpans, Slovenia
EXF-171	E	Human; *Keratomycosis*; Brasil
EXF-2682	F	Human; *Trichomycosis nigra*; Italy
EXF-10513	G	Deep sea water; Italy
EXF-151	H	Human; *Tinea nigra*; Portugal
EXF-6651	I	Spider web; Atacama desert; Chile
EXF-6669	J	Spider web; Atacama desert; Chile
EXF-6654	K	Spider web; Atacama desert; Chile
EXF-6656	L	Rock wall in a cave; Atacama desert; Chile

The properties of the sequenced genomes were comparable in all major aspects, except in the size and number of predicted genes, which were much lower in strains C and D (Table 2). The average genome size of the other strains was 48.51 Mbp (SD ±1.43 Mbp), and the average number of predicted genes was 16,658 (SD ±568), while in case of strains C and D the genome sizes were 25.2 Mbp and 25.3 Mbp, and the numbers of predicted genes were 8690 and 8674, respectively. In all cases the genomes and predicted proteomes contained more than 97% of identifiable fungal Benchmarking Universal Single-Copy Ortholog genes/proteins; in genomes C and D all of them were single-copy, while in other genomes more than 60% were duplicated (Additional file 1: Table S1). Genomes C and D were thus considered to be haploid and other genomes diploid, and this classification was confirmed by further analyses (as described below).

**Table 2:** Statistics of sequenced *H. werneckii* genomes

Statistic	A^a^	B	C	D	E	F	G	H	I	J	K	L
Coverage	–	35×	34×	34×	26×	25×	33×	28×	23×	26×	28×	28×
Genome assembly size (Mb)	49.9	49.4	25.2	25.3	45.2	48.1	50.5	49.2	48.6	48.5	48.4	48.7
Number of contigs	651	1568	447	425	1679	2042	5740	4192	5073	5127	4335	4146
Contig N50	153,735	89,304	183,917	238,279	100,620	83,336	17,360	25,995	19,686	19,095	24,150	24,982
CDS total length (Mb)	24.14	27.07	13.71	13.70	24.31	25.84	24.45	24.98	24.54	24.41	25.10	24.91
CDS total length (% of genome)	48.38%	54.82%	54.42%	54.26%	53.81%	53.68%	48.44%	50.78%	50.47%	50.29%	51.91%	51.13%
Gene models (*n*)	15,974	17,329	8690	8674	15,340	16,354	17,094	16,775	16,845	16,712	16,886	16,583
Number of exons (*n*)	38,282	38,881	19,797	19,586	37,036	38,738	36,837	36,596	36,685	37,384	37,202	38,880
Exons per gene (average)	2.40	2.24	2.28	2.26	2.41	2.37	2.15	2.18	2.18	2.24	2.20	2.34
GC content (%)	53.50%	53.58%	53.35%	53.43%	53.25%	53.25%	53.40%	53.10%	53.16%	53.16%	53.22%	53.16%

^a^*Data for reference genome A were adapted from Sinha* et al [2].

**Fig. 1:**
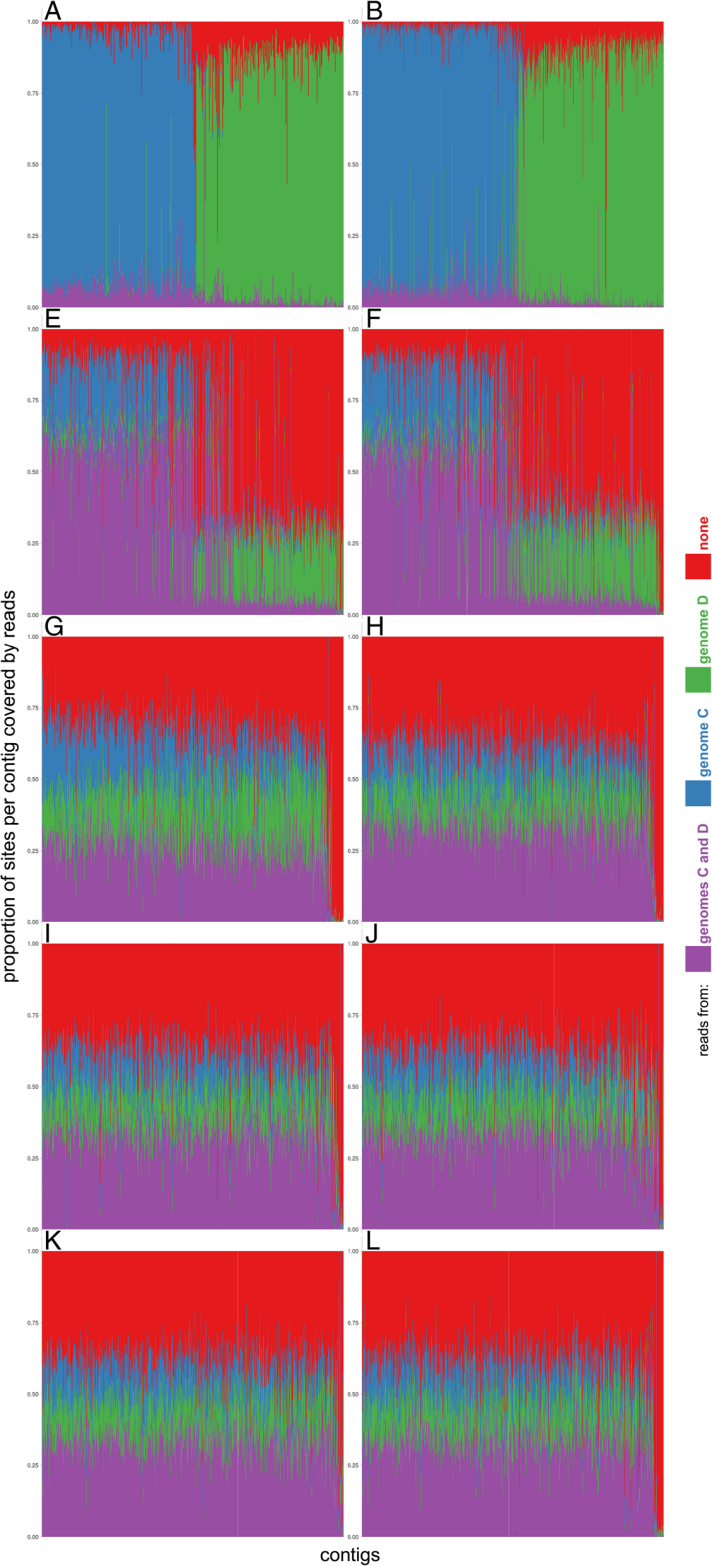
Overlap between mapping of reads from haploid *H. werneckii* genomes C and D to diploid genomes of *H. werneckii*. Each stacked bar represents one contig of a diploid genome and shows the proportion of sites within the contig covered by reads from genomes C (blue), D (green), both (purple) or none (red). Plot labels (A-L) correspond to the genome names. Contigs shorter than 20 kBp were omitted from the analyses

Mapping of sequencing reads of haploid strains C and D to all other (assembled and diploid) genomes resulted in approximately half of the diploid genomes remaining uncovered by reads. Three distinct mapping patterns were observed (Fig. 1, Additional file 1: Figures S1–S3). In case of genomes A and B, reads from strain C mapped well to approximately half of each genome, while reads from strain D mapped to the other half. The same phenomenon was observed in the alignment of the assembled genomes of strains A, C and D (Additional file 1: Figure S4) due to close relatedness of haploid strains with different subgenomes of the diploid reference strain, as discussed below. In case of genomes E and F reads mapped well to approximately one half of the genome, but in this case the overlap between the mappings of C and D reads was much larger (Fig. 1, Additional file 1: Figures S1–S3). In other genomes the mapping was relatively uniformly distributed across the genome.

**Fig. 2:**
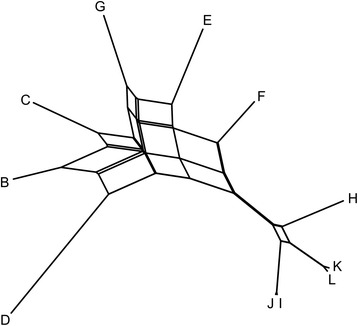
Phylogenetic network of sequenced *H. werneckii* strains. The network was reconstructed with the Neighbor-Net algorithm based on the dissimilarity distance matrix calculated from the SNP data

Using the previously published reference genome A as the reference for subsequent variant calling, a satisfactory mapping of sequencing reads could not be achieved. Only between 32 and 54% of reads could be uniquely mapped for strains E-L, while for strains B, C and D the proportion of uniquely mapping reads was much higher (71–92%) (Additional file 1: Table S2). Therefore the mapping and variant calling was repeated using the haploid genome C as the reference and the resulting SNP data were used for the reconstruction of the phylogenetic network. On the network strain B is positioned between the haploid strains C and D (Fig. 2). Strains H-J were furthest from the haploid strains, while the remaining strains E-G were located in the intermediary positions. Substantial reticulation was observed, especially between the strains B-G.

As observed previously in the case of the reference *H. werneckii* genome [2], high heterozygosity was observed within other diploid strains of the species as well. The proportion of diploid genomes that could be uniquely aligned between the two subgenomes ranged from 67.30% for genome E to 93.66% for genome G, while the share of identical nucleotides in the aligned regions was between 88.31 and 91.92% (Additional file 1: Table S3).

**Fig. 3:**
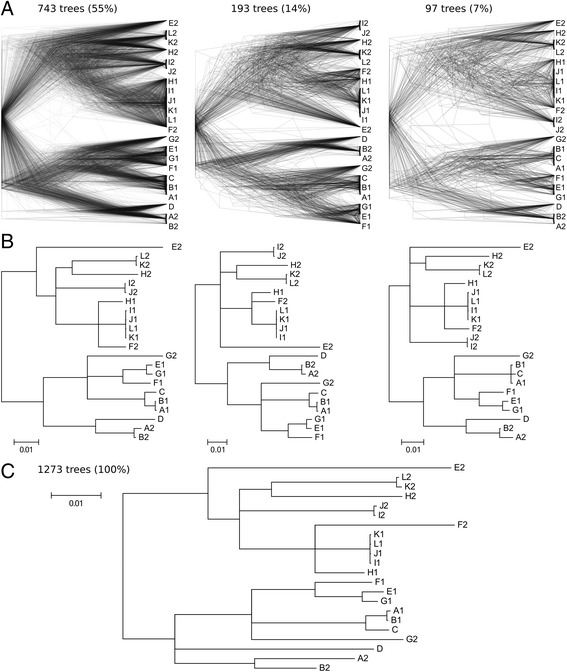
Phylogenetic trees of 1273 core genes present in a single copy in haploid genomes and in two copies in diploid genomes. The trees were clustered by similarity (with minimum normalized Robinson-Foulds at 0.80). Trees from three largest clusters (together representing 76% of all trees) are shown as an overlay (**a**) and as strict consensus trees (showing only nodes occurring more than 50% of the time) (**b**). A strict consensus tree for all core genes is shown in panel (**c)**

To investigate the phylogenies of separate subgenomes of diploid *H. werneckii* strains, core genes present in exactly two copies in the diploid and exactly one copy in the haploid strains were used as proxies for the subgenomes. Phylogenies of 1273 core genes were largely concordant, with three largest clusters of similar trees containing 76% of all trees (Fig. 3). Phylogenetic lineages connecting paralogous genes from the same strains were consistent between the genes. Two large phylogenetic lineages could be distinguished, with both paralogs from the same strain consistently belonging to the same lineage with the exception of the strains E and F (Fig. 3), which were also the most heterozygous (Additional file 1: Table S3). Almost the same topology was observed when phylogeny was inferred using taxonomically relevant genes: RNA polymerase II (RPB2) and beta tubulin genes (Additional file 1: Figure S5) although one copy of RPB2 could not be found in genome E and one copy beta tubulin gene copy was missing from the assembled genomes B and H.

The possible evidence for sexual reproduction was analyzed through linkage disequilibrium. The index of association between the loci was calculated in ten randomly sampled subsets of 1000 SNPs. The null hypothesis of absent linkage (related to sexual reproduction) was rejected for all subsets with *p* < 0.001, indicating that *H. werneckii* is clonal (Additional file 1: Figure S6).

**Fig. 4:**
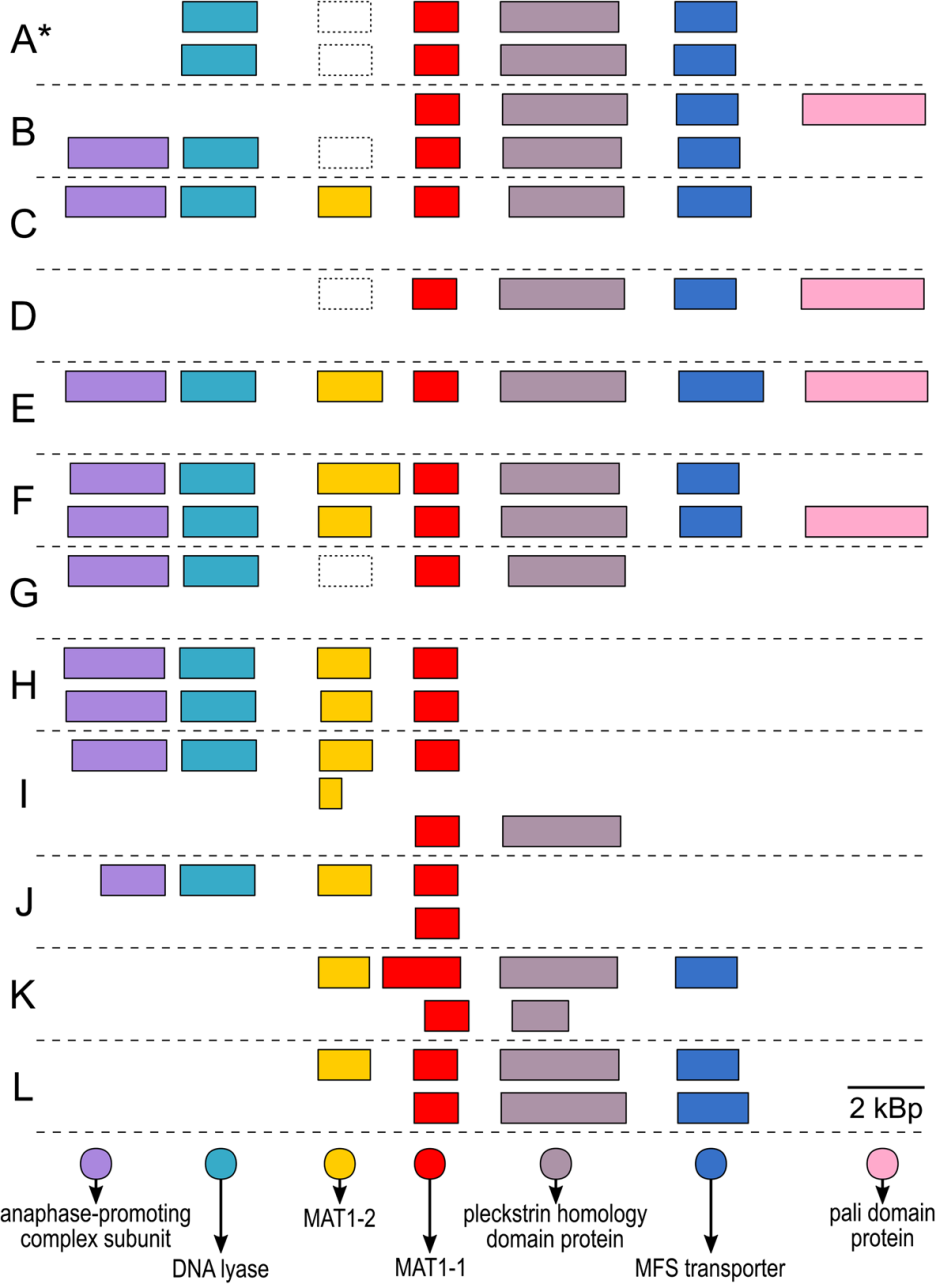
Putative mating loci in different strains of *Hortaea werneckii*. Short hypothetical coding regions that were inconsistently annotated in individual genomes are not shown. MAT1–1: mating type-1; MAT1–2: mating type-2. Putative MAT1–2 pseudogenes are marked with dashed rectangles. *Reference strain mating locus adapted from [1]

Putative mating loci have been identified in all sequenced strains (Fig. 4). In genomes A, B, D and G no genes were predicted in the region where MAT1–2 gene was expected. When this region was manually searched against the GenBank database for proteins with BLASTX, no matches were found. However, when these genomic regions were compared to the well-conserved MAT1–2 genes from other *H. werneckii* strains, the similarity was clearly recognizable, suggesting they are homologous, although the protein sequence identity in the alignable regions was only 70–80% and the translated proteins contained frameshifts. In all other strains at least one homothallic mating locus was identified (but several were incomplete due to truncated contigs).

## Discussion

Sequencing of eleven genomes of the black yeast *Hortaea werneckii* provides us with new insights into the evolutionary history of the unusual duplicated genomes of this species.

Reference-guided assembly and annotation of the genomes showed that nine of them were similar in size (the average difference from the reference genome size, 49.9 Mbp, was less than 3%; Sihna et al. [2]), number of predicted proteins, GC content and other characteristics (Table 2). The assemblies and predicted proteomes can be considered as almost complete (Additional file 1: Table S1). In contrast, genomes of strains C and D were clear outliers. The genome assembly and gene count of these two strains are half the size of the genomes of the reference strain and other strains. Also, while in other strains more than 60% of Benchmarking Universal Single-Copy Ortholog genes/proteins were found in two copies, no duplicated genes/proteins were found in the assembled genomes C and D and their predicted proteomes (Additional file 1: Table S1). The strains C and D were thus considered to be haploid, and this classification was supported by all subsequent analyses. Two thirds of reads from eight strains’ genomes to the reference genome could not be uniquely aligned to a single locus of the reference genome (Additional file 1: Table S2). This can be explained by the diploid nature of the reference genome and the resulting failure of the mapping algorithm to map reads to only one of the duplicated loci. However, in case of three strains (B-D) the proportion of uniquely mapping reads was much higher, reflecting the closer phylogenetic relatedness of these strains to the reference strain (Fig. 2).

Additional insight into the relationship between the haploid and diploid strain genomes was provided by mapping the reads of the haploid genomes (C and D) against assembled genomes of diploid strains, resulting in three mapping patterns (Fig. 1, Additional file 1: Figures S1–S3): (i) Reads from each haploid strain mapping to a different subgenome within the genomes A and B, indicating that these diploid genomes are hybrids of ancestors of strains C and D, as also demonstrated by the alignment of assembled genomes C and D to the reference genome A (Additional file 1: Figure S4); (ii) Reads from haploid strains mapping with higher affinity to the same half of the genomes of strains E and F due to a closer relatedness of both C and D to one of the subgenomes within strains E and F (Fig. 3); (iii) Relatively homogenous mapping throughout the genomes, likely due to a larger (and equal) phylogenetic distance between the genomes of strains C and D and both subgenomes of each of the diploid strains G-L.

**Fig. 5:**
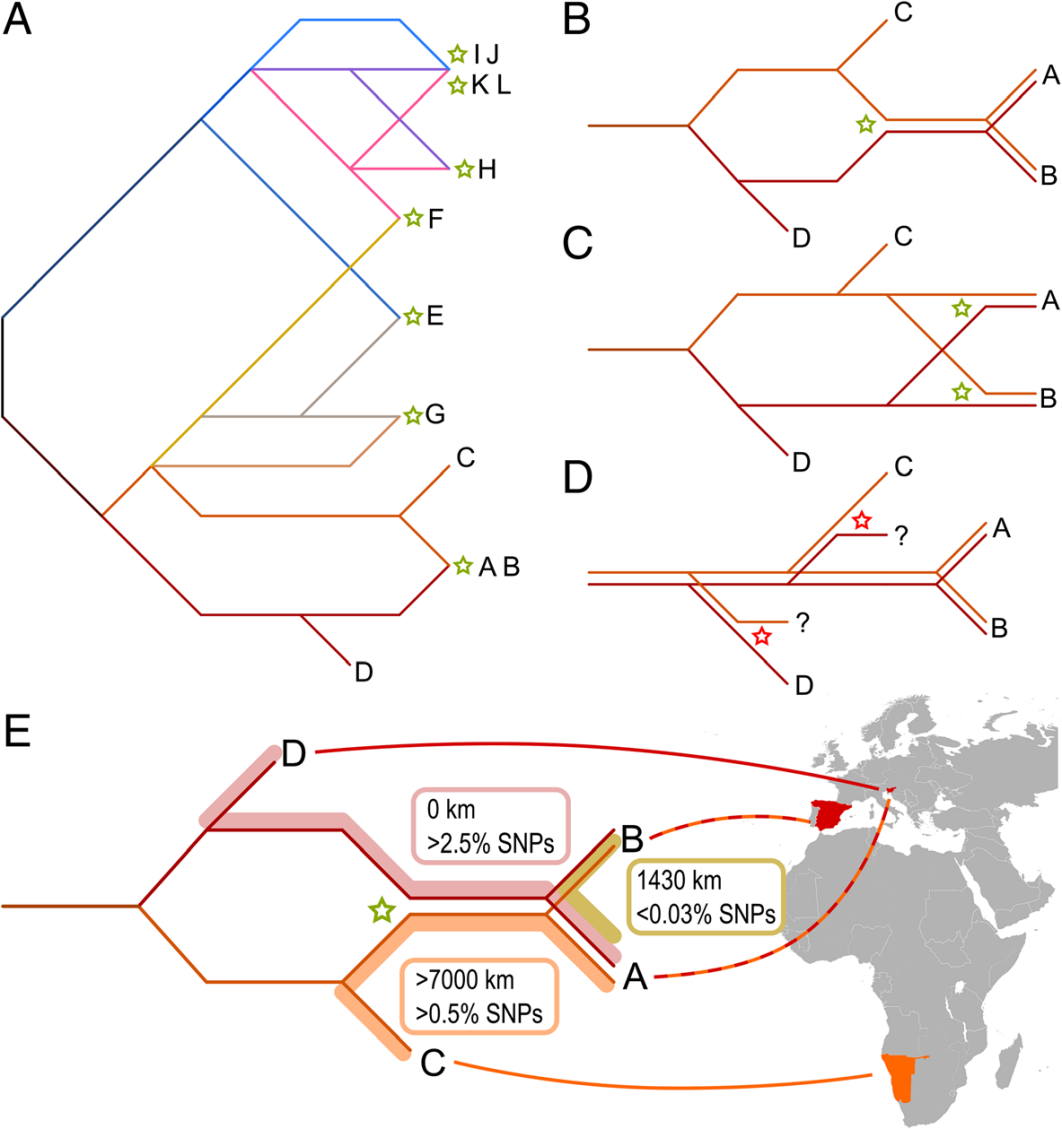
A scheme of the hybridization events, as indicated by the phylogenies of core genes, producing *H. werneckii* genomes investigated in the study (panel **a**), and the possible series of events connecting haploid strains C and D and diploid strains A and B (panels **b, c, d**), and selected geographical and phylogenetic distances between genomes A-D (panel **e**). Each line represents a haploid genome. Putative hybridization events are marked with green stars, putative haploidization events are marked with red stars (only in panels **b**, **c**, **d**)

SNP based phylogeny (Fig. 2) and gene phylogenies (Fig. 3) confirmed these interpretations and supported the following conclusions:

1. Comparative genomics and the discovery of two haploid *H. werneckii* strains do not support the endoreduplication hypothesis, which was previously proposed as the most likely cause of the reference genome duplication [2]. Instead, the almost exclusive alignment of genomes of the two haploid strains C and D each to a different half of the reference genome (Fig. 1) as well as the gene phylogenies (Fig. 3) indicate that the reference *H. werneckii* strain is a hybrid of ancestors of C and D (Fig. 5). The same hybridization event likely led to strain B (Fig. 5b). This hypothesis is in agreement with the observation that many autopolyploid species are relatively heterozygous, suggesting that they were created by intraspecific hybridization rather than endoreduplication [6]. The proposed hybridization between strains with around 10% nucleotide differences in the alignable genomic regions (Additional file 1: Table S3) is also in line with the mating between parents whose genomes differ by 4–15% nucleotides that was observed in *Candida metapsilosis*, *C. orthopsilosis* and *Zygosaccharomyces* sp. (reviewed by Ortiz-Merino et al. [8]). Two separate hybridization events for genomes A and B from closely related haploid strains also explain our observations (Fig. 5c). A diploid ancestor producing strains C and D through haploidization (Fig. 5d) is improbable unless the haploid genomes in the cells coexist without genome unification, e.g. by delaying the nuclear fusion and can thus be perfectly separated during the haploidization. In an ascomycetous yeast this would be unusual and preliminary microscopic observations detected only one nucleus per *H. werneckii* cell (M. Lenassi et al., personal communication). Haploidization can also be achieved through gene loss, but in this scenario the resulting haploid genome is also expected to be a mix of both parental subgenomes.

2. Genomes of strains G-L are a result of hybridization events distinct from the event producing genomes of strains A and B. While a more detailed investigation of the evolutionary history of these genomes will be difficult to perform unless their corresponding haploid genomes are identified, phylogenies of genes representing the duplicated genomes (Fig. 3, Additional file 1: Figure S5) can be best explained by six hybridizations in addition to the one producing the reference genome (Fig. 5a). One of the hybridization parents of strains E and F was much more closely related to the haploid genomes C and D, explaining the preferential mapping of these haploids to only approximately half of the genomes of strains E and F (Fig. 1). The level of heterozygosity within the diploid strains matched these inferred phylogenetic distances between the subgenomes, which were shortest in the genome G and largest in strains E and F (Additional file 1: Table S3).

3. Although comparative genomics points to several hybridization events in the evolution of analyzed *H. werneckii* strains, recombination between different phylogenetic lineages within the species appears to be rare. Apart from one hybridization event there appears to have been no major exchange of genetic material in the phylogenetic lineages connecting genomes A and B on the one hand and the genomes C and D on the other hand, despite the large geographical and/or phylogenetic distances between these strains (Fig. 5). Any such exchange would disrupt the almost perfect separation of the reference genome into two subgenomes, each more closely related to either strain C or strain D. Interestingly, few to no large scale losses within the subgenomes occurred after hybridization, each still representing approximately half of the duplicated genome (as noted already by Sinha et al. [2]). The high degree of concordance between gene trees (Fig. 3, Additional file 1: Figure S5) also points to little (if any) exchange of genetic material between the investigated phylogenetic lineages (apart from the few hybridization events) and fulfils the “strong phylogenetic signal” criterion for clonality [10]. Clonality is also indicated by the linkage disequilibrium analysis, which rejected the null hypothesis of sexuality in *H. werneckii*.

A largely clonal evolutionary history of *H. werneckii* interspersed by occasional hybridization events is well described by the concept of restricted recombination, which claims that in clonal species recombination is not necessarily completely absent, but is rather rare enough not to disrupt the prevalent clonal population structure pattern [10]. Considering the prevalence of mating loci in *H. werneckii* genomes (Fig. 4) it is difficult to explain the apparent absence of sexual reproduction. The presence of a mating locus, however, is not sufficient for mating [9]. Many restrictions to recombination are possible, both extrinsic and intrinsic, from limited dispersal and population bottlenecks, skewing the balance of mating types (*H. werneckii* can be found in sea water, but is by far the most abundant in hypersaline environments, which are often well-isolated from each other) to hybrid incompatibility [9]. Also, the limited (and thus potentially biased) selection of strains sequenced in this study might not have been sufficient to detect recombination. For example, the yeast *Cryptococcus gattii* can appear clonal if sampled locally, but is recombining on a global scale [11].

When the reference *H. werneckii* genome was first sequenced, the mating locus was identified as heterothallic. However, we here showed that in all strains the mating locus was at least initially homothallic, although the MAT1–2 may have degenerated into a pseudogene in strains A, B, D and G and due to poor conservation could not be identified as such in the original investigation of the reference genome. This does not exclude the possibility of mating as the mechanism of hybridization in the lineage leading to genomes A and B. In both haploids MAT1–1 is well conserved and haploid C also has a conserved MAT1–2. Even if only MAT1–1 remained functional in the ancestors of strains A and B, sexual reproduction between strains of the same mating type (unisexuality) has been observed in fungi before [12]. Another possible mechanism of hybridization is parasexuality, fusion of vegetative cells, in this case not followed by a mitotic or meiotic haploidization, yielding a persistent diploid.

Several questions related to the diploid *H. werneckii* genome remain to be addressed by further sampling, sequencing and studies of physiology. Can diploid strains revert to haploids? Considering the strong evidence for clonality and a lack of an obvious mechanism that could split a diploid genome back into parent haploid genomes this does not appear to happen often if at all. An alternative option is the persistence of haploid strains on the evolutionary scale, and occasional hybridization of these haploids into persistent diploids. Additionally, it is not clear how ancient are the hybridization events. Strains I-L isolated from the same cave in Atacama share one subgenome but not the other, suggesting that at least these hybridizations might have occurred relatively recently in a geographically limited area and sharing one of the parents.

Finally, if as it appears here, the hybridization events do not significantly contribute to recombination in the species, does the unusual ploidy of *H. werneckii* play a role in its extremotolerant physiology? The osmotolerant *Pichia sorbitophila* is a known example of a species formed by (in this case intraspecies) hybridization [13]. Our initial investigations have not identified any substantial physiological differences between haploid and diploid strains (unpublished data), but further research is needed to either confirm or reject these preliminary results.

## Conclusions

Comparative genomics of eleven strains of the black yeast *H. werneckii*, in the past extensively studied for its extreme halotolerance, showed that the majority of investigated strains were diploid, arising from several hybridization events between relatively heterozygous ancestors in a species that otherwise appears to be limited to clonal reproduction. This, together with the first identification of haploid *H. werneckii* strains establishes the species as a good (extremotolerant) fungal model for studying the effects of ploidy and hybridization on the evolution of the genome in a system largely unperturbed by sexual reproduction and genetic recombination between the phylogenetic lineages.

## Methods

### Cultures, media and growth conditions

Eleven *Hortaea werneckii* strains collected from various habitats around the world (Table 1) were obtained from the Ex Culture Collection of the Department of Biology, Biotechnical Faculty, University of Ljubljana (Slovenia). They were selected to represent different parts of the intraspecific phylogeny as estimated by the standard phylogenetic markers (data not shown) and with no prior knowledge about their ploidy. Cultivation of biomass for the isolation of DNA was performed in the standard chemically defined medium Yeast Nitrogen Base (YNB, Qbiogene), with 0.5% ammonium sulphate (*w*/*v*), and 2% glucose (w/v). The pH was adjusted to 7.0 prior to autoclaving. 2% of agar (w/v) were added for solid media. All cultures were grown at 24 °C. Liquid cultures were grown on a rotary shaker at 180 rpm. Cells were harvested in the mid-exponential growth phase (OD_600_ = 0.8–1.0) with centrifugation (10 min at 5000×g), the pellet was frozen in liquid nitrogen and kept at − 80 °C until DNA isolation.

### DNA isolation

DNA for sequencing was isolated from the prepared biomass. The frozen pellet was first homogenized using a pestle and mortar. 100 mg of the homogenate was transferred to 2 ml microcentrifuge tubes, each with one stainless steel ball, placed in holders pre-cooled with liquid nitrogen and additionally homogenized in Retsch Mixer Mill 301 (Thermo Fisher Scientific, USA) at 20 Hz for 1 min. 300 μl MicroBead Solution buffer was added and the mixture was completely thawed on ice. This homogenate was then used for DNA extraction using the UltraClean Microbial DNA isolation kit (MO BIO Laboratories, USA) according to the manufacturer instructions. Contaminating RNA was removed with RNAse A (Thermo Fisher Scientific, USA). The quantity, purity and integrity of the isolated DNA was evaluated by agarose electrophoresis, spectrophotometrically with NanoDrop 2000 (Thermo Fisher Scientific, USA) and by Qubit fluorometry (Thermo Fisher Scientific, USA).

### Genome sequencing

The sequencing was performed by GATC Biotech AG (Germany) on Genome Sequencer Illumina HiSeq with 2× 150 bp Nextera libraries in a multiplexed mode. The resulting output was demultiplexed, the quality was checked with FastQC and the reads were trimmed for adaptors and quality (Q20 threshold) with the bbduk script (https://jgi.doe.gov/data-and-tools/bbtools/).

Sequencing reads, assembly and annotation data have been deposited in Genbank under the BioProject PRJNA428320 and in Open Science Framework 97 (10.17605/OSF.IO/HQWXG).

### Variant calling

Mapping of reads to non-haploid genomes is a non-trivial task that has to be optimised on a case-by-case basis [14]. Mapping with bwa mem [15] to the published reference genome of *H. werneckii* (GenBank MUNK00000000.1) [2] was tested here with different combinations of parameter values: (i) default values; (ii) discarding reads mapping to more than one locus (using options “-c” and “-r” followed by removing all mappings flagged with “XA:Z:” or “SA:Z:”; all of this with different thresholds for lowest reported alignment score (option “-T”: 30 (default), 60, 90, 95).

After genome C was determined to be haploid, the mapping was repeated with this genome used as the reference. Due to the lack of comparable reads the genome A was excluded from this analysis. Mapped reads were sorted with samtools 1.6 [16], and duplicates marked with picard 2.10.2. Variant calling was performed with Genome Analysis Toolkit 3.8 [17] according to “GATK Best Practices” with the “hard filtering” option. Ploidy was set to 2 for diploid strains and 1 for haploids. The most stringent filtering criterion was depth of coverage. Aligning highly heterozygous diploid genomes to the haploid reference is associated with a risk of aligning only reads of one subgenome, but not the other (due to excessive dissimilarity of these reads from the reference), resulting in underestimation of the heterozygosity. Thus only reads with a high depth of coverage were used for the subsequent analyses (Additional file 1: Figure S7).

The density of genomes coverage by sequence reads was calculated by samtools 1.6 [16] and visualized in R with ggplot2 [18, 19].

### Assembly and annotation

The genomes were assembled with IDBA-Hybrid 1.1.3 [20] with the published *H. werneckii* genome [2] used as a reference to guide the assembly process. The maximum k value selected was 180, minimum support in each iteration was 2, similarity for alignment 0.95, seed kmer 20, maximum allowed gap in the reference was 100 and the minimum size of contigs was 500.

Annotation of protein-coding and tRNA genes was performed with MAKER 2.31.8 [21]. The fungal subset of the Swissprot database (recovered on 19. 7. 2017) and the published predicted proteome of *H. werneckii* [2] were used as evidence. Three ab initio gene predictors were used in the MAKER pipeline. Semi-HMM-based Nucleic Acid Parser (SNAP) [22] was bootstrap-trained within MAKER based on the gene models derived from the alignment of the protein datasets to the genome as recommended by Campbell et al. (2014). GeneMark-ES (Lomsadze et al., 2014) was self-trained [23] and Augustus was used with the training parameters for *Neurospora crassa* [24].

The genome assembly and gene prediction completeness was evaluated with the Benchmarking Universal Single-Copy Orthologs (BUSCO 3) software [25] in genomic and proteomic modes, using the dataset for fungi [26]. The genomic mode was used with augustus trained on the genome of *Neurospora crassa*. All other parameters were left at default values.

Pairwise alignments of genomes A, C and D (discarding all contigs shorter than 25 kBp) were calculated with the nucmer algorithm, as implemented in Mummer 3.23, and plotted with the mummerplot utility [27] as described by Hane et al. [28].

The differences between the subgenomes of diploid *H. werneckii* strains (as a measure of heterozygosity within the genomes) were calculated by separating the diploid genomes into two subgenomes as described by Sinha et al. [2] and pairwise aligning the subgenomes with the nucmer algorithm of Mummer 3.23 [27] using anchor matches unique in both the reference and query. The alignment was summarized with the show-coords algorithm (Mummer 3.23) using default parameters and the resulting table was analyzed with R [18] to count the proportion of the genome covered by the alignments and the average share of identical nucleotides in the alignments.

### Phylogenetic analyses

Phylogenetic network was reconstructed from SNP data called using genome C as the reference (as described above). The dissimilarity distance matrix was calculated by the R package poppr [29] and used to construct the phylogenetic network with the Neighbor-Net algorithm as implemented in the R package phangorn [18, 30].

Gene phylogenetic trees were constructed from predicted coding sequences of all here sequenced genomes and the reference genome. First, BLAST clustering (1e-40 e-value threshold) and analysis of alignments (with 80% identical nucleotides threshold) were used to identify CDSs existing in exactly two copies in diploid genomes and in one copy in haploid genomes using the stand-alone BLAST+ 2.7.1 [31] and processing of the results with a custom script. Sequences from each resulting CDS cluster were aligned with MAFFT 7.215 with the “--auto” option and default parameters [32], the alignment was optimized with Gblocks 0.91 using options “-b3=10 -b4=3 -b5=n” [33] and used for the reconstruction of phylogeny with PhyML 3.1 [34] if it was longer than 200 nucleotides and contained on average at least 15 nucleotide differences between the gene pairs. Hasegawa-Kishino-Yano, 85 [35] nucleotide substitution model was used, and alpha parameter of the gamma distribution of substitution rate categories and the proportion of invariable sites were estimated by PhyML. Finally, the trees were sorted into clusters of trees with similar topology measured by the normalized Robinson-Foulds distance calculated by the ETE Toolkit 3.1.1 [36]; the minimum similarity within the cluster was 0.80. The largest clusters of trees were visualized with DensiTree 2.2.5 [37] and a strict consensus tree was calculated for each cluster with the consensus_tree.py script in QIIME, using only nodes occurring more than 50% of the time [38].

Phylogenies of RNA polymerase II and beta tubulin genes were estimated by automatically aligning the nucleotide sequences with MAFFT [32], estimating the custom model of nucleotide substitution with jModelTest 2.1.10 [39] and generating the phylogenetic tree by PhyML 3.1 [34]. The alpha parameter of the gamma distribution of substitution rate categories and the proportion of invariable sites were estimated by PhyML. Branch supports were estimated with aLRT as Chi2 based supports.

### Linkage disequilibrium and mating type loci

Linkage disequilibrium was estimated by calculating the index of association *r*_*d*_ [40] using the package poppr in R [18, 29]. The index was calculated on ten datasets, each containing 1000 randomly sampled SNPs. The *p*-value for the rejection of the null hypothesis (that the loci are not linked and the population is sexual) was estimated with 999 permutations of each dataset.

Mating genes were identified by BLAST searches against the assembled *H. werneckii* genomes and predicted proteomes, using homologues from other dothideomycetous fungi as queries. Annotated genomes were used to identify the flanking genes. The function of the resulting predicted proteins was inferred by blast comparison with the most similar proteins in the GenBank database.

## Additional files

Additional file 1: Contains figures illustrating the mapping of haploid genomes C and D to diploid genomes of *H. werneckii* (**Figures S1-S4**), phylogenetic trees of RNA polymerase II and beta tubulin genes (**Figure S5**), the result of the index of association test of sexuality/clonality (**Figure S6**) and an illustration of how the single nucleotide polymorphisms were filtered by depth of coverage (**Figure S7**). The file also contains the results of the search for Benchmarking Universal Single-Copy Orthologs in *H. werneckii* genomes (**Table S1**), the results of mapping of sequencing reads to the reference *H. werneckii* genome (**Table S2**) and the results of the alignment of genomic regions within the same diploid genomes (**Table S3**). (PDF 6665 kb)
