# Supplementary material for: Genomic evidence for intraspecific hybridization in a clonal and extremely halotolerant yeast
Source: BMC Genomics. 2018 May 15;19:364. doi: 10.1186/s12864-018-4751-5 (PMC5952469; doi:10.1186/s12864-018-4751-5)
Supplement: Supplementary file 1 — Contains figures illustrating the mapping of haploid genomes C and D to diploid genomes of H. werneckii (Figures S1-S4), phylogenetic trees of RNA polymerase II and beta tubulin genes (Figure S5), the result of the index of association test of sexuality/clonality (Figure S6) and an illustration of how the single nucleotide polymorphisms were filtered by depth of coverage (Figure S7). The file also contains the results of the search for Benchmarking Universal Single-Copy Orthologs in H. werneckii genomes (Table S1), the results of mapping of sequencing reads to the reference H. werneckii genome (Table S2) and the results of the alignment of genomic regions within the same diploid genomes (Table S3). (PDF 6665 kb) [file 12864_2018_4751_MOESM1_ESM.pdf]

Supplementary File to the article:
**Genomic evidence for intraspecific hybridization in a clonal and extremely halotolerant yeast**
by
**Cene Gostinčar, Jason E. Stajich, Jerneja Zupančič, Polona Zalar, Nina Gunde-Cimerman**

Published in: BMC Genomics

**Figure S1. Mapping of sequencing reads from strains C (upward pointing histograms) and D (downward pointing histograms) to assembled diploid *H. werneckii* genomes.** The mapping depth was calculated as a moving median in a 1000 nt window for all contigs larger than 2500 nt.

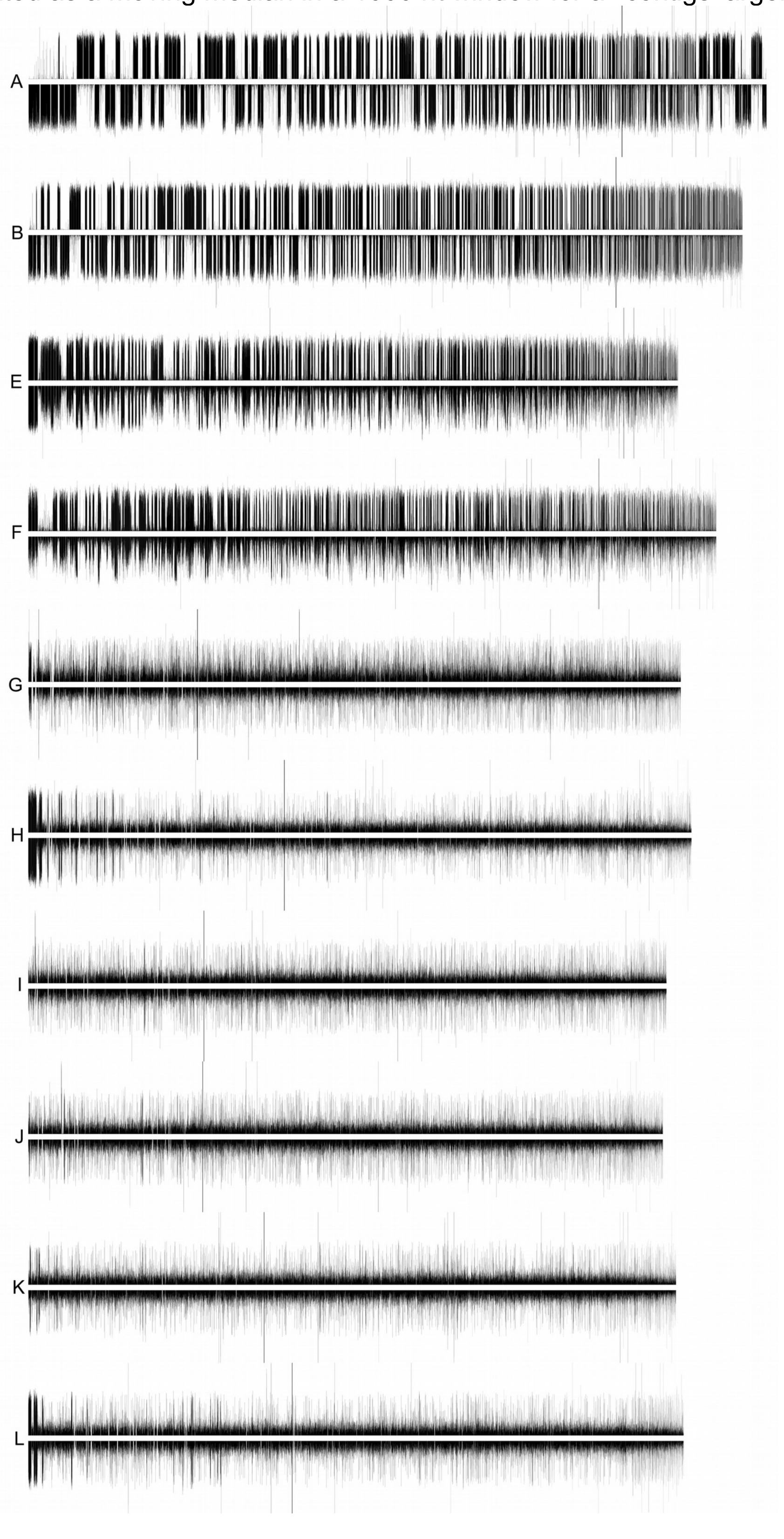

**Figure S2. Number of sites with specific coverage by reads from haploid *H. werneckii* genomes C and D in diploid *H. werneckii* genomes.** The histograms show the frequency of sites with coverage from 0 to 100. The number of sites with zero coverage from either genome is represented by a blue column. The red curves show the cumulative frequency of sites with specific coverage. Dashed blue lines mark the diploid (upper line) and haploid (lower line) genome sizes.

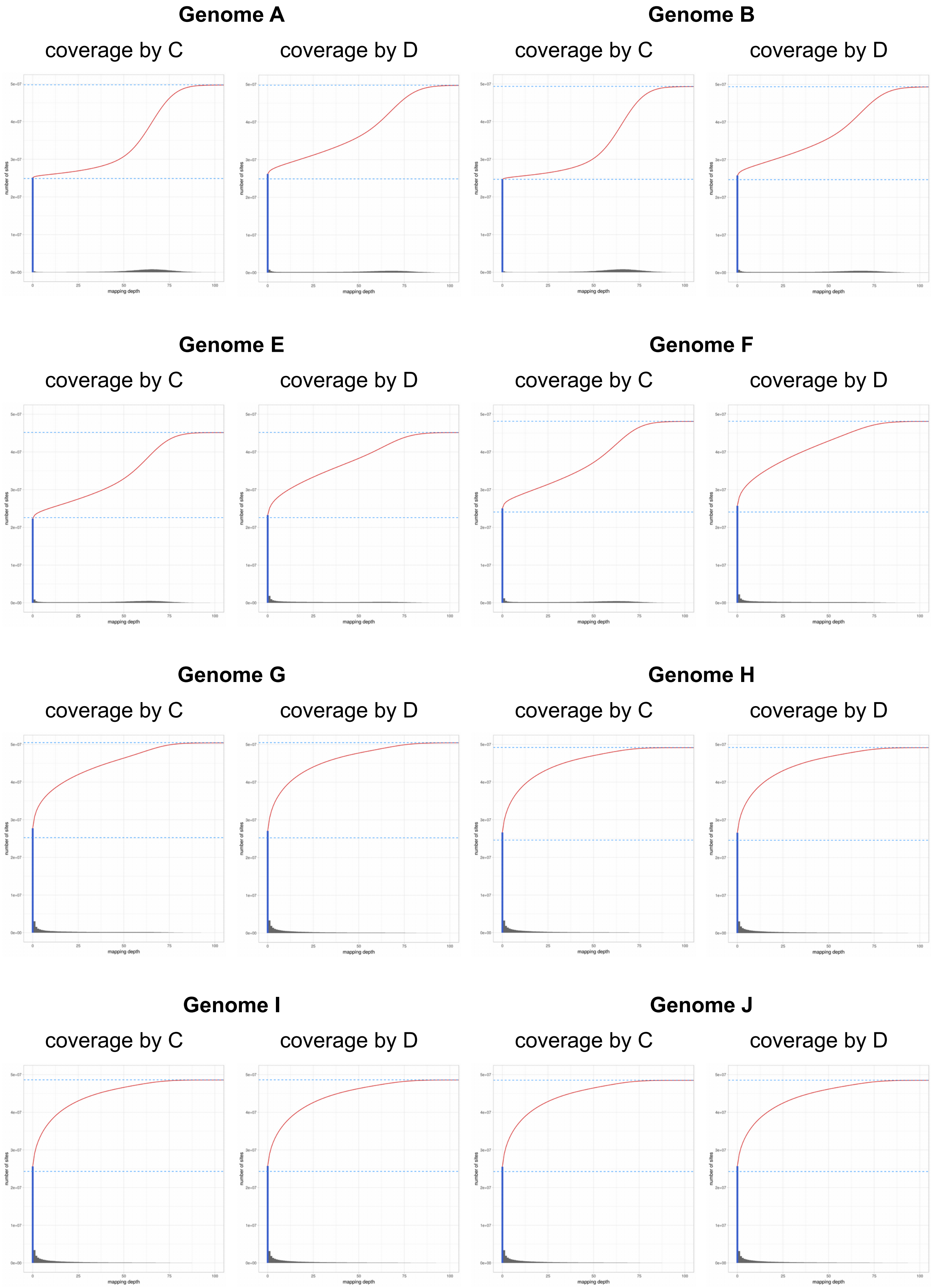

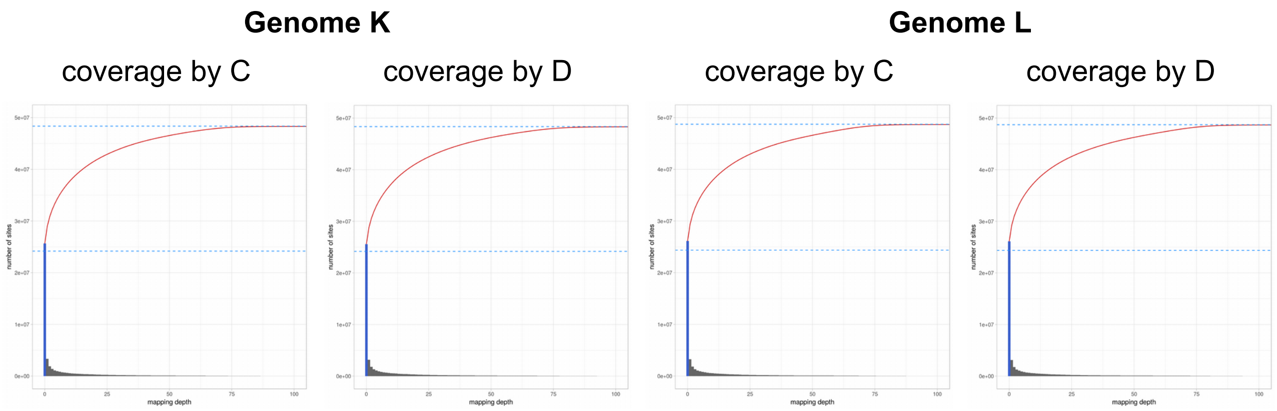
Genome K
Genome L
coverage by C
coverage by D
coverage by C
coverage by D
number of sites
mapping depth
0e+00
1e+07
2e+07
3e+07
4e+07
5e+07
0
25
50
75
100

**Figure S3. Number of sites with summarized coverage by reads from haploid *H. werneckii* genomes C and D in diploid *H. werneckii* genomes.** The red histograms show the frequency of sites with summarized coverage by reads from both C and/or D. The yellow histograms show only sites covered by reads from *both* C *and* D. The numbers of sites with zero coverage from either genome are represented by blue columns. Red and yellow curves show the cumulative frequencies corresponding to red and yellow histograms, respectively. Dashed blue lines mark the diploid (upper line) and haploid (lower line) genome sizes.

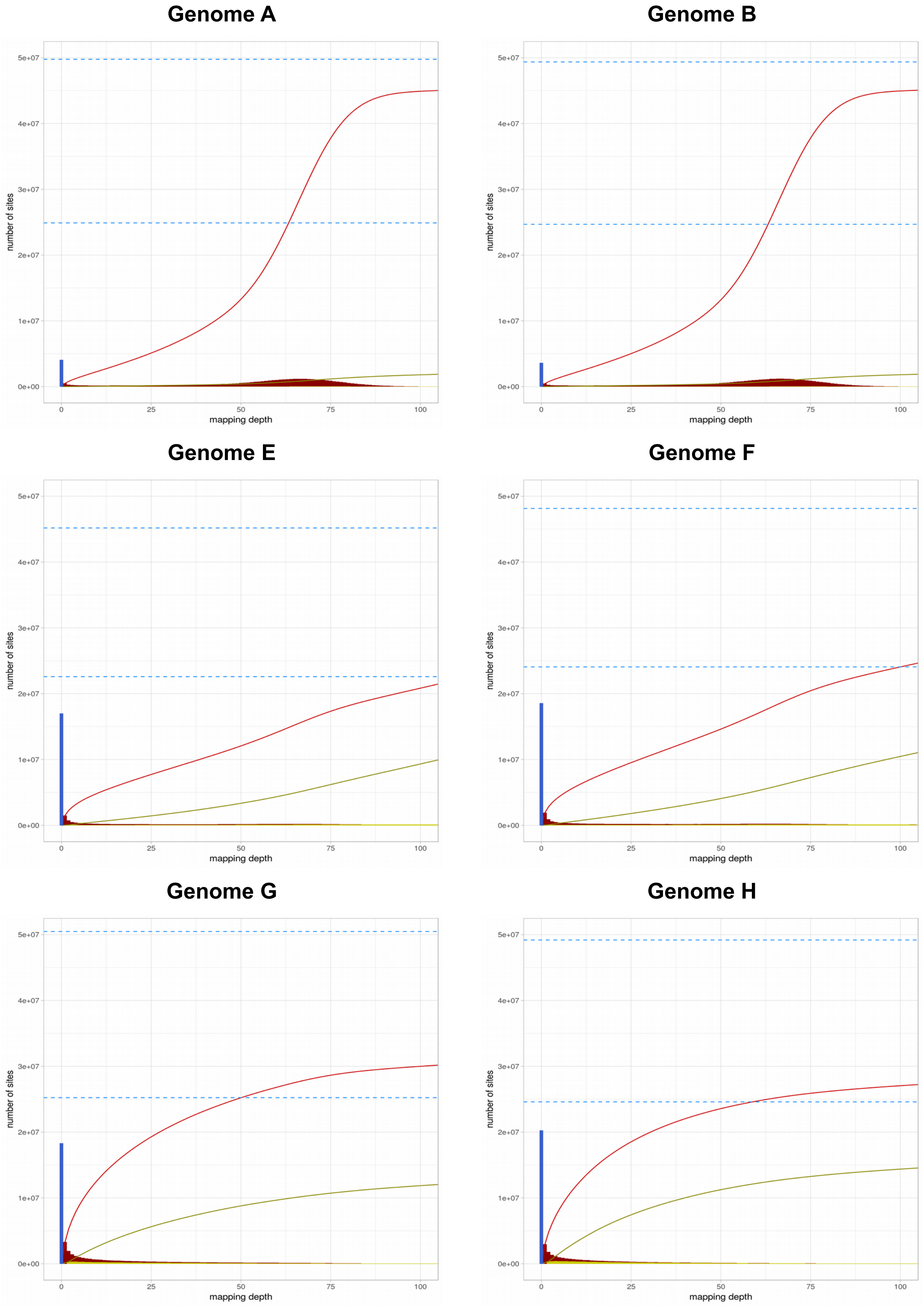

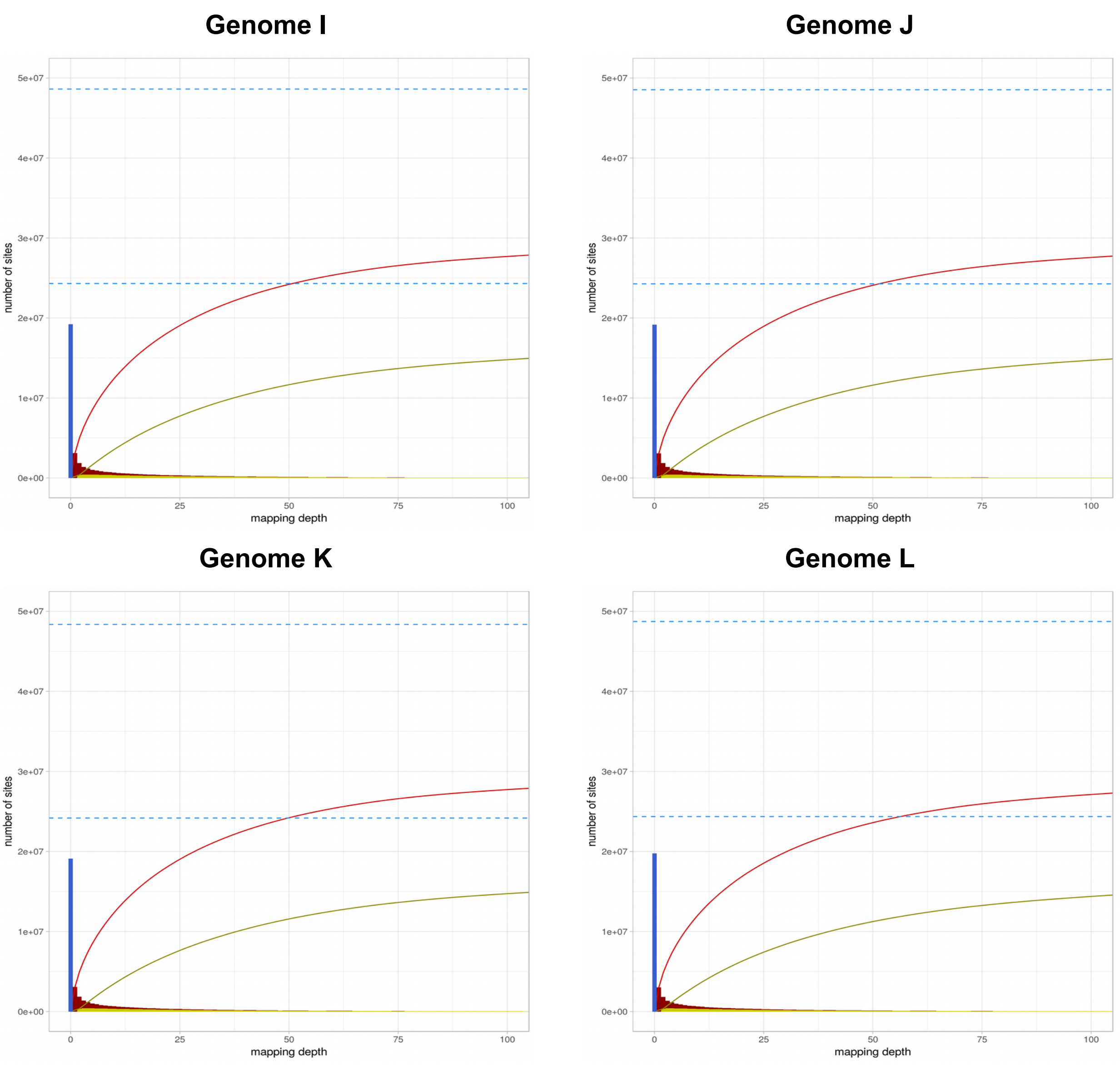
Genome I
Genome J
Genome K
Genome L
number of sites
mapping depth
5e+07
4e+07
3e+07
2e+07
1e+07
0e+00
0
25
50
75
100

**Figure S4. Alignment of assembled genomes of strains C and D to the reference genome A.** Only contigs longer than 25 kBp and best aligning regions are shown.

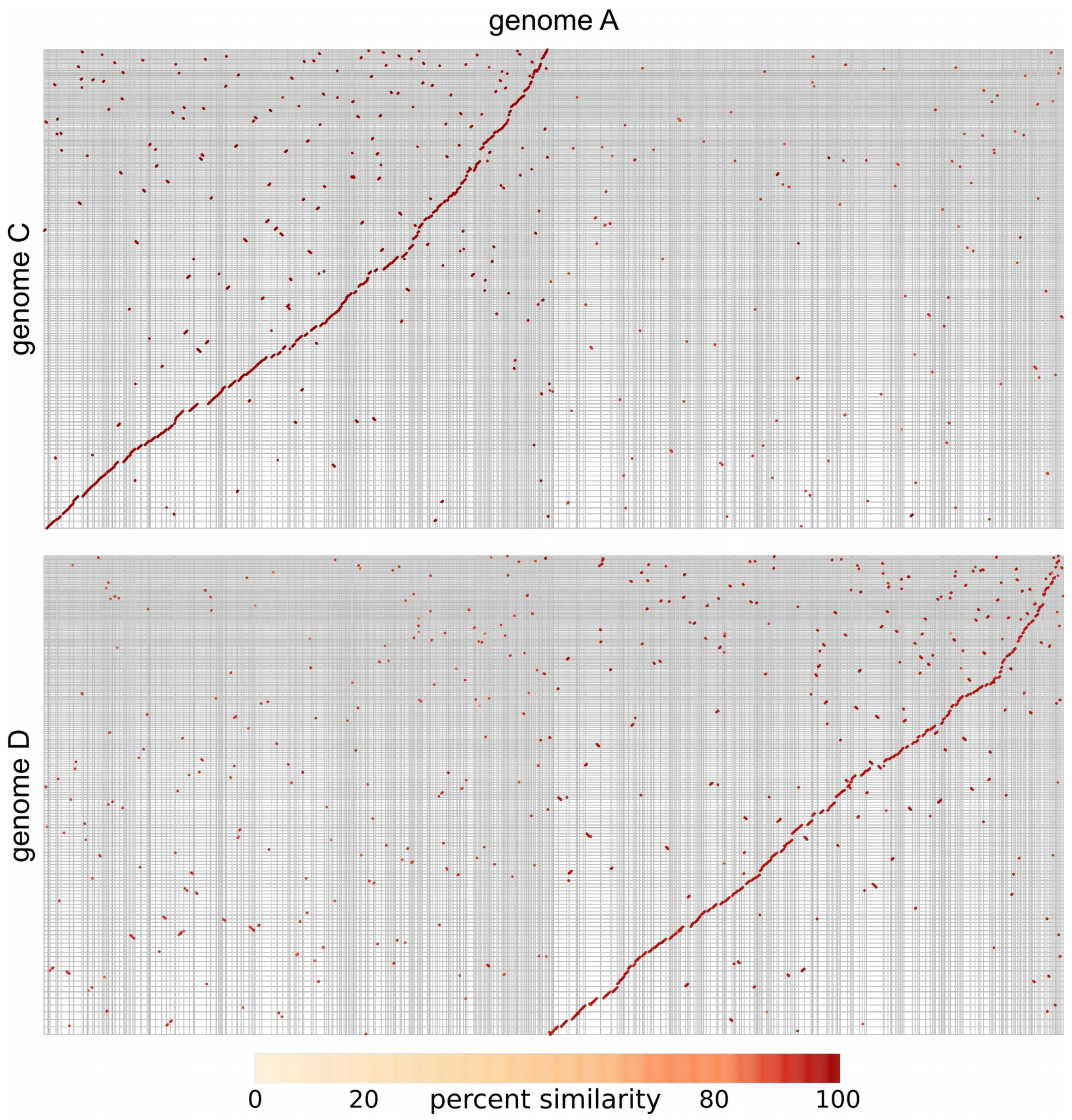

**Figure S5. Phylogeny of taxonomic markers.** Maximum likelihood phylogenies based on genes encoding RNA polymerase II and beta tubulin in twelve strains of *H. werneckii*. The consensus phylogeny based on 1273 phylogenies of core genes (left; as shown in Fig. 4) is provided for comparison.

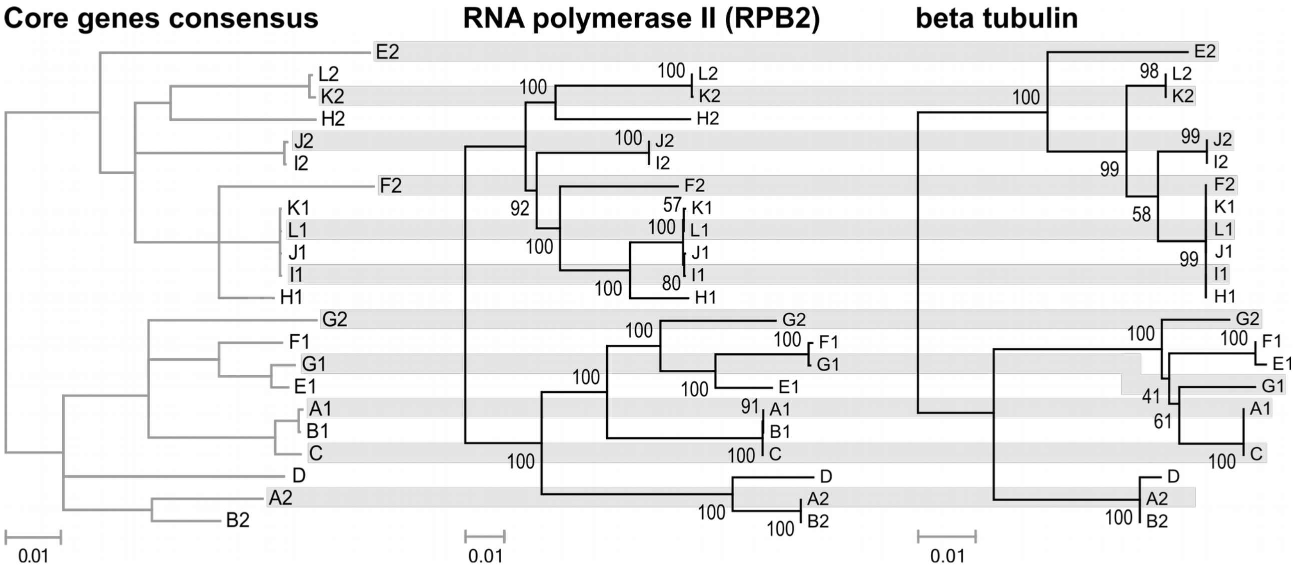


**Figure S6. Test of sexuality/clonality.** The index of association $\bar{r}_d$ was calculated on ten random sampled data subsets (the figure is representative for all of them), each containing 1000 SNPs. 999 permutations were used to estimate the p-value for the rejection of the null hypothesis (that the loci are not linked and the population is sexual). The observed index of association is marked with the vertical dashed blue line, while the expected indices for non-linked (permutated) data are shown as a gray histogram. Data corrected for clonality produced the same result.

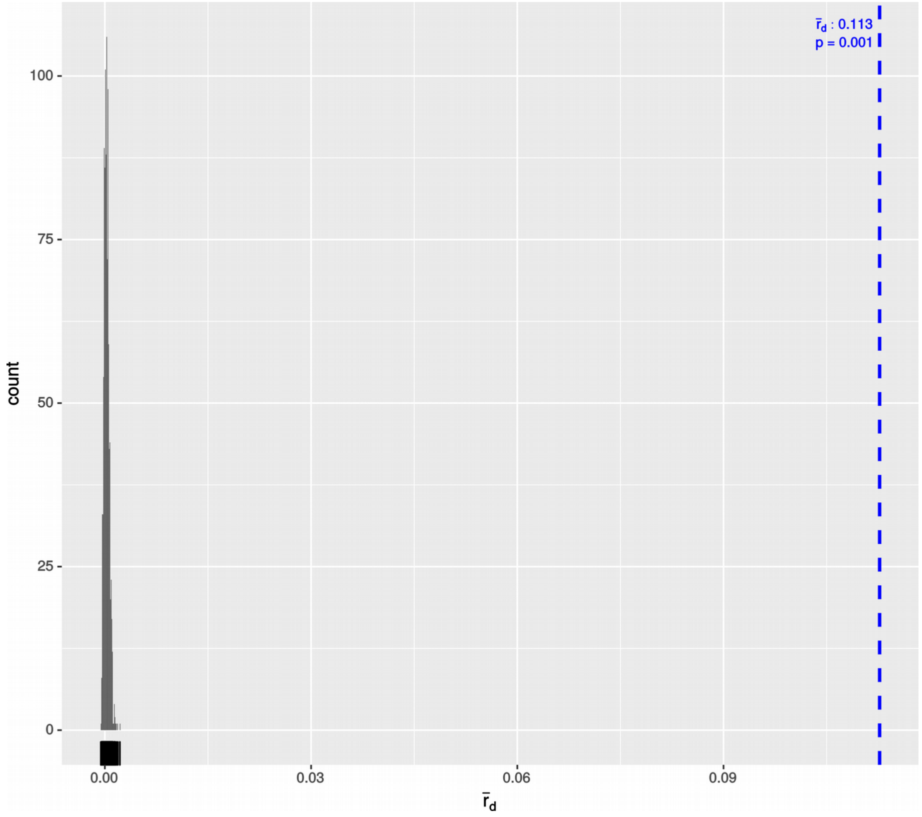

**Figure S7. Filtering of SNP data by depth of coverage.** To avoid the underestimation of heterozygosity due to mapping of sequencing reads from only one of the two subgenomes within a diploid genome, all variants with low coverage were removed in the filtering step of the variant calling pipeline. SNPs between the blue and red vertical lines were included in the final dataset.

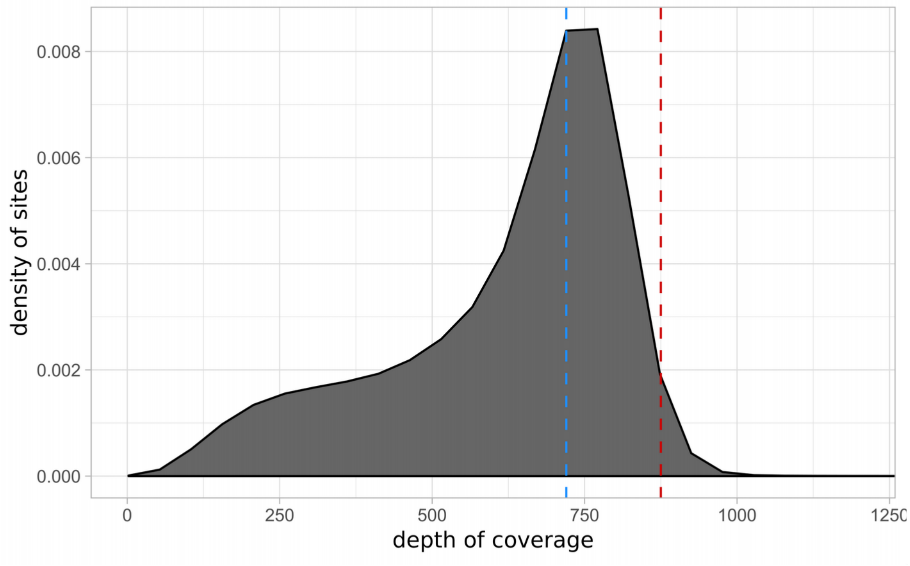

**Table S1**. Numbers of identified Benchmarking Universal Single-Copy Orthologs in the assembled genomes and predicted proteomes of *H. werneckii*, demonstrating high levels of completeness of the genomes and proteomes and a large proportion of duplicated genes/proteins in diploid strains.

| **Genome** | **B** | **C** | **D** | **E** | **F** | **G** | **H** | **I** | **J** | **K** | **L** |
|---|---|---|---|---|---|---|---|---|---|---|---|
| Complete BUSCOs | 278 | 282 | 283 | 282 | 282 | 255 | 269 | 267 | 268 | 269 | 274 |
| Complete and single-copy BUSCOs | 12 | 282 | 283 | 69 | 43 | 76 | 72 | 66 | 87 | 62 | 62 |
| Complete and duplicated BUSCOs | 266 | 0 | 0 | 213 | 239 | 179 | 197 | 201 | 181 | 207 | 212 |
| Fragmented BUSCOs | 7 | 2 | 3 | 6 | 4 | 28 | 17 | 20 | 16 | 16 | 11 |
| Missing BUSCOs | 5 | 6 | 4 | 2 | 4 | 7 | 4 | 3 | 6 | 5 | 5 |
| Total BUSCO groups searched | 290 | 290 | 290 | 290 | 290 | 290 | 290 | 290 | 290 | 290 | 290 |

| **Predicted proteome** | **B** | **C** | **D** | **E** | **F** | **G** | **H** | **I** | **J** | **K** | **L** |
|---|---|---|---|---|---|---|---|---|---|---|---|
| Complete BUSCOs | 278 | 278 | 281 | 277 | 275 | 258 | 263 | 263 | 264 | 265 | 269 |
| Complete and single-copy BUSCOs | 21 | 278 | 281 | 68 | 45 | 81 | 66 | 69 | 88 | 61 | 60 |
| Complete and duplicated BUSCOs | 257 | 0 | 0 | 209 | 230 | 177 | 197 | 194 | 176 | 204 | 209 |
| Fragmented BUSCOs | 9 | 6 | 6 | 9 | 10 | 25 | 21 | 19 | 21 | 21 | 17 |
| Missing BUSCOs | 3 | 6 | 3 | 4 | 5 | 7 | 6 | 8 | 5 | 4 | 4 |
| Total BUSCO groups searched | 290 | 290 | 290 | 290 | 290 | 290 | 290 | 290 | 290 | 290 | 290 |

**Table S2**. Percent of reads mapped to the reference *H. werneckii* genome (genome A) with the bwa aligner including and excluding reads aligning to more than one locus and using different minimum thresholds (T) for the alignment score.

| **strain** | | **uniquely aligning reads with different alignment score thresholds (T)** | | | |
|---|---|---|---|---|---|
| | **default bwa parameters** | **T=30** | **T=60** | **T=90** | **T=95** |
| **B** | 99% | 92% | 92% | **92%** | 92% |
| **C** | 98% | 87% | 87% | **87%** | 87% |
| **D** | 94% | 72% | 71% | **71%** | 71% |
| **E** | 86% | 47% | 46% | 45% | 45% |
| **F** | 85% | 45% | 44% | 43% | 43% |
| **G** | 88% | 54% | 53% | 52% | 52% |
| **H** | 79% | 35% | 34% | 33% | 33% |
| **I** | 81% | 36% | 34% | 33% | 33% |
| **J** | 82% | 35% | 34% | 33% | 33% |
| **K** | 81% | 36% | 34% | 33% | 33% |
| **L** | 78% | 34% | 33% | 32% | 32% |

**Table S3**. Proportion of genomes that could be uniquely aligned to homologous regions within the same genome of diploid *H. werneckii* strains and the average nucleotide identity within the alignments.

| **Genome** | **A** | **B** | **E** | **F** | **G** | **H** | **I** | **J** | **K** | **L** |
|---|---|---|---|---|---|---|---|---|---|---|
| % genome aligning within the genome | 82.45 | 81.42 | 67.30 | 71.43 | 93.66 | 86.19 | 87.37 | 87.74 | 87.09 | 84.57 |
| % identity of aligned regions | 89.38 | 89.04 | 88.31 | 88.40 | 91.92 | 91.58 | 91.87 | 91.86 | 91.66 | 91.64 |
